# Clinical Performance of Zirconia Veneers Bonded with MDP-Containing Polymeric Adhesives: A One-Year Randomized Controlled Trial

**DOI:** 10.3390/polym17091213

**Published:** 2025-04-29

**Authors:** Viet Anh Nguyen, Truong Nhu Ngoc Vo, Minh Son Tong, Thi Nhu Trang Nguyen, Thu Tra Nguyen

**Affiliations:** 1 School of Dentistry, Hanoi Medical University, Hanoi 10000, Vietnam; nhungoc@hmu.edu.vn (T.N.N.V.); tongminhson@hmu.edu.vn (M.S.T.); nhutrang@hmu.edu.vn (T.N.T.N.); nguyenthutra@hmu.edu.vn (T.T.N.); 2 Faculty of Dentistry, Phenikaa University, Hanoi 12116, Vietnam

**Keywords:** zirconia laminate veneers, polymeric adhesive systems, MDP monomer, clinical performance, resin-based cement

## Abstract

Acid-etched zirconia has emerged as a high-strength alternative to traditional glass ceramics for laminate veneers in aesthetic dentistry. This randomized, double-blind controlled clinical trial aimed to evaluate the one-year clinical performance of zirconia veneers etched with a hydrofluoric-nitric acid mixture and bonded using a 10-methacryloyloxydecyl dihydrogen phosphate (MDP) containing polymeric adhesive system, compared to lithium disilicate veneers. Fifty-two patients were treated with either translucent zirconia or lithium disilicate veneers, and restorations were bonded using light-cured resin-based adhesives. Clinical parameters, including veneer survival, esthetics, marginal adaptation, postoperative sensitivity, and periodontal health, were assessed using modified United States Public Health Service (USPHS) criteria and periodontal indexes at 2 weeks, 6 months, and 12 months. Both materials showed high survival rates with no statistically significant differences in clinical outcomes. One zirconia veneer debonded early but was successfully rebonded without fracture, while one lithium disilicate veneer fractured upon debonding. The findings support the viability of acid-etched zirconia veneers bonded with polymer-based adhesives as a durable and esthetic restorative option. The study highlights the clinical relevance of polymeric bonding systems in enhancing zirconia veneer performance and reinforces their role in modern adhesive dentistry.

## 1. Introduction

Ceramic laminate veneers are minimally invasive prosthetic restorations with proven excellent aesthetics and long-term clinical success rates [1,2]. Traditionally, laminate veneers have been fabricated from glass ceramics, such as feldspathic porcelain, leucite-reinforced feldspathic porcelain, and lithium disilicate. The glass content in these materials allows for translucency and etchability, enabling strong bonds with resin-based adhesives due to the solubility of silica in hydrofluoric acid. However, their low crystalline content results in reduced flexural strengths, making them susceptible to fracture during try-in and post-bonding cracks [3]. Furthermore, dark tooth substrates are difficult to mask with these ceramics due to their high glass content. Moreover, the fabrication processes of glass ceramic veneers, including refractory die, platinum foil, and heat-pressing techniques, are complicated and costly. Recent advancements in computer-aided design and computer-aided manufacturing (CAD/CAM) technology have enabled the fabrication of ceramic laminate veneers through milling or three-dimensional (3D) printing [4]. Despite these advancements, the cost of ceramic 3D printers and wet milling machines for partially sintered glass ceramics remains high.

To overcome the above disadvantages of glass ceramics, ceramics with high crystalline content, such as yttria partially stabilized zirconia, have been utilized to fabricate laminate veneers [5]. Zirconia restorations are generally fabricated with dry milling, a more cost-effective method than wet milling. Zirconia-based ceramics exhibit high flexural strength due to transformation toughening, a process where the tetragonal phase transforms into the monoclinic phase under stress [6]. This transformation increases volume, effectively halting crack propagation. Additionally, the high crystalline content blocks light transmission, resulting in opacity that can mask discolored tooth structure. On the other hand, this opacity can be a disadvantage when aiming for a natural tooth appearance. The introduction of translucent zirconia, achieved by modifying the zirconia structure to increase yttria and cubic phase percentages, has improved translucency by allowing more light transmission [7,8]. This enhanced translucency, however, comes at the cost of reduced flexural strength due to a decrease in the tetragonal phase.

Another disadvantage of zirconia-based ceramics is their inability to be etched with standard hydrofluoric acid due to the absence of silica. This is particularly significant for laminate veneers, a type of bonded restoration that lacks the macro-mechanical retention found in full-coverage crowns. The retention of bonded restoration depends primarily on the adhesive bond between the ceramic and the tooth structure. Sandblasting is a common surface treatment method used to create micro-mechanical retention and enhance bonding with resin-based adhesives on zirconia [9]. However, this technique can lead to the formation of microcracks and increase the monoclinic phase, potentially reducing the flexural strength of zirconia [10]. To overcome these limitations, a recently developed alternative involves treating the zirconia surface with a mixture of hydrofluoric and nitric acids. This technique modifies the zirconia surface at a microstructural level, enhancing its chemical affinity to polymer-based resin adhesives. In particular, adhesives containing 10-methacryloyloxydecyl dihydrogen phosphate (MDP), a functional monomer with a strong affinity for metal oxides, have demonstrated superior bonding performance in vitro [11,12].

Although zirconia veneers have been investigated in earlier reports, the majority of published studies are limited to case reports or a single randomized controlled trial (RCT), which involved multiple veneers per patient [5,13,14]. Such designs may simplify shade matching but do not fully reflect the clinical challenges encountered in single-unit restorations, where harmonizing color and translucency with adjacent natural teeth is significantly more demanding, especially under minimal thickness constraints typical of veneer preparation.

Currently, only one clinical study has evaluated the clinical outcomes of zirconia laminate veneers treated with sandblasting, while limited data exist regarding zirconia veneers bonded using chemical etching protocols and advanced polymeric adhesive systems [13]. To address this gap, the present randomized controlled clinical trial was designed to assess the one-year clinical performance of single-unit zirconia laminate veneers etched with a hydrofluoric–nitric acid mixture and bonded using an MDP-containing light-cured polymeric resin cement. Lithium disilicate veneers served as the positive control. To the best of our knowledge, this is the first RCT to evaluate acid-etched zirconia veneers under standardized conditions. The study’s null hypothesis proposed that there would be no significant difference in the marginal fit and one-year clinical performance between zirconia and lithium disilicate veneers.

## 2. Materials and Methods

### 2.1. Study Design

**Figure 1:**
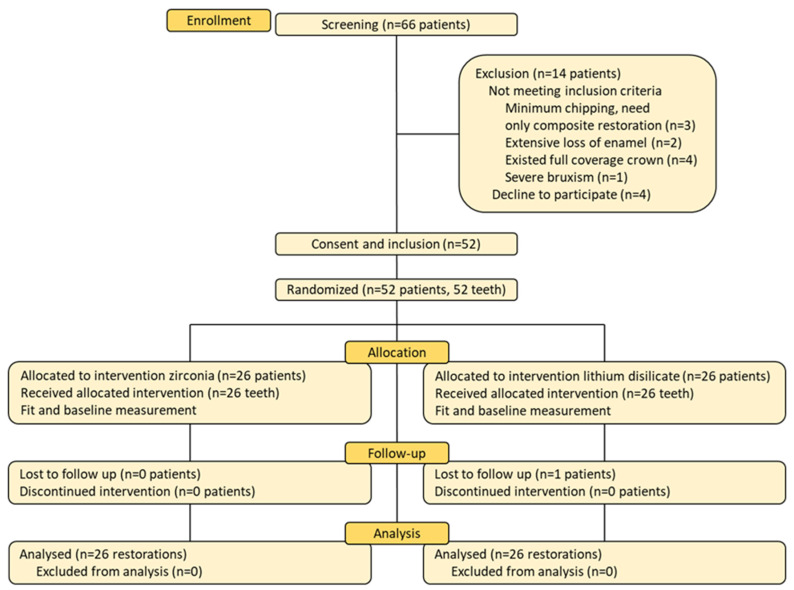
The CONSORT study flowchart.

This double-blinded, parallel-group, randomized clinical trial was conducted at the High Technical Center of Dentistry, School of Dentistry, Hanoi Medical University. The present protocol was submitted and approved (4/2023) by the Institutional Ethical Review Board of the Hanoi Medical University, Hanoi, Vietnam (approval No 01050122106) and registered at clinicaltrials.gov (NCT06406582). The sample size calculation was based on a three-year success rate of zirconia crowns at 30.2% in the study of Gherlone et al., with an alpha level of 0.05 and a power of 0.8 [15]. The calculated sample size was 21 patients per group. However, a total of 52 participants were actually recruited, with 26 participants in each arm. Details of participant allocation and follow-up are presented in a CONSORT (Consolidated Standards of Reporting Trials) study flowchart (Figure 1).

The study participants consisted of adult patients undergoing prosthodontic treatment. Recruitment was performed from April 2023 to March 2024 by a certified prosthodontist at the Department of Prosthodontics. Eligible patients were recruited if they satisfied all the inclusion criteria and did not exhibit any of the exclusion criteria. Each participant was enrolled in the study once, with a single tooth receiving intervention, to isolate the effect of the treatment on that specific tooth, avoiding clustered outcomes influenced by individual patient characteristics affecting multiple restorations [1]. Eligible patients were informed and counseled about the study, and they read and signed informed consent forms. Efforts were made to achieve adherence by explaining the minimally invasive nature of ceramic laminate veneers.

Inclusion criteria comprised adult patients aged 18–90 years at the time of prosthodontic treatment, from both genders, with one maxillary or mandibular permanent incisor, canine, or premolar indicated for restoration with ceramic laminate veneers. These indications include spacing, discoloration, malformation, endodontic treatment, chipping or fracture, and mild crowding. Exclusion criteria will consist of minimal chipping that only requires enameloplasty or composite restoration, tooth bruxism or other bad oral habits harming restorations, short teeth, inadequate enamel for bonding purposes, existing large restorations, and inadequate supporting tooth structure.

The randomization was performed by a researcher who was not involved in patient recruitment, laboratory restoration fabrication, or clinical management using a randomizing function of a spreadsheet program (Excel 2016, Microsoft, Redmond, WA, USA). Then, the researcher prepared and placed the randomization list into opaque envelopes. After tooth preparation for laminate veneers, the restoration was assigned to one of two groups on the day of laboratory fabrication, with the envelope containing the computer-generated randomization list opened by the dental laboratory technician. The zirconia group (ZV) served as the intervention group, in which ceramic laminate veneers were fabricated from translucent zirconia crystalline ceramics (Nacera, Doceram GmbH, Dortmund, Germany). The lithium disilicate group (LDV) served as the positive control group, in which ceramic laminate veneers were manufactured from lithium disilicate glass ceramics (IPS e.max, Ivoclar, Schaan, Liechtenstein).

An equal number of patients were assigned to the intervention and control groups. The randomization was independent of tooth types, indications, and prepared abutment forms. Both the patients, clinician, and outcome assessor were blinded to the group assignments. The dental technician was not blinded due to the differing manufacturing processes.

### 2.2. Intervention

**Figure 2:**
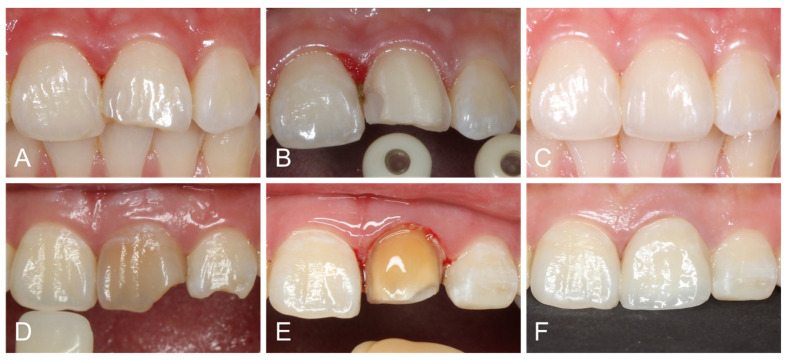
(**A**) Initial view of fractured central incisor. (**B**) Veneer preparation without breaking interproximal contact points. (**C**) Lithium disilicate veneer after 2 weeks. (**D**) Initial view of discolored and endodontically treated central incisor. (**E**) Veneer preparation with proximal breakage. (**F**) Zirconia veneer after 2 weeks.

A single clinician prepared the teeth for ceramic laminate veneers, using calibrated guiding depth grooves (Dia-Burs, Mani, Tochigi, Japan) on the labial surface and incisal edge. The preparation included approximately 0.5–0.7 mm of labial reduction and 1.0–1.5 mm of incisal/occlusal reduction with a butt joint incisal finish line [16]. A chamfer finish line was prepared on the cervical and interproximal regions, extending approximately 0.5 mm subgingivally. Endodontically treated premolars were prepared using a veneerlay design, which involved both occlusal and labial surface reduction. Dentin exposure was kept as minimal as possible to preserve enamel for bonding purposes. However, in cases of dark abutments, more preparation was performed to provide enough space for adequate masking. The proximal contact was not broken in most cases, except for diastema, existing large proximal restorations, or dark abutments (Figure 2). Silicone indexes were used to evaluate the reduction thickness throughout the preparation process. The abutments underwent rounding of all angles and thorough polishing.

After preparation, digital impressions of the upper arch, lower arch, and occlusion were taken with an intraoral scanner (Omnicam, Dentsply Sirona, Bensheim, Germany). Temporary restorations were fabricated chairside with composite resin (Filtek, 3M, Seefeld, Germany). The scan data was sent to the laboratory to manufacture ceramic laminate veneers with CAD/CAM milling technology.

The laminate veneers were milled from translucent zirconia discs or lithium disilicate blocks, with shades carefully matched to adjacent teeth, guided by shade tabs (3D Master Shade Guide, VITA, Säckingen, Germany). The milled veneers were sintered according to the manufacturers’ guidelines. Zirconia veneers were etched with a hydrofluoric acid-nitric acid mixture (ZIRCOS-E, Eunjin Chemical, Gunsan, Republic of Korea) for two hours, while lithium disilicate veneers were etched with 9% hydrofluoric acid for 20 s (Porcelain Etch, Ultradent, South Jordan, UT, USA) [11,12]. For maxillary anterior teeth with high aesthetic demands, the laminate veneers were cut back to provide space for characterization using layered feldspathic porcelain (Ceramco 3, Dentsply, Konstanz, Germany) of fluorapatite ceramics (IPS e.max Ceram, Ivoclar, Schaan, Liechtenstein). The veneer thickness was measured at the incisal/occlusal and labial areas with a caliper.

At the bonding appointment, the temporary restorations were removed, followed by thoroughly cleaning the abutments with non-fluoride pumice. The laminate veneers were then tried on the abutments to evaluate their contour, shade, and marginal adaptation. Next, the marginal and internal fit of laminate veneers was recorded with the silicone replica method [17,18]. This involved filling the gap between the veneers and abutments with a light-body silicone impression material (Honigum Light, DMG, Hamburg, Germany) and then capturing the thin, polymerized layer with a heavy-body silicone (Honigum Heavy, DMG, Hamburg, Germany).

**Figure 3:**
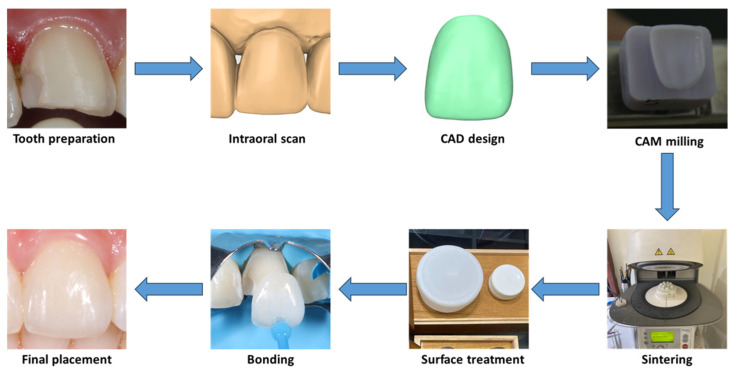
Digital workflow for veneer fabrication and cementation.

Subsequently, all the ceramic veneers were cleaned in an ultrasonic bath with 90% alcohol for 10 min. Next, the abutments were isolated with rubber dams and etched with 37% phosphoric acid. The bonding procedure was then performed with a universal primer containing 10-MDP (Single Bond Universal, 3M, Seefeld, Germany) and a light-cured veneer resin adhesive (RelyX Veneer LC, 3M, Seefeld, Germany) (Figure 3). After adhesive polymerization, the excessive resin was removed with a No. 12 scalpel blade (KIATO, Kanpur, India), followed by a delicate margin finishing and polishing with silicone cups and diamond paste. Finally, occlusal adjustments were carried out in the maximum intercuspation, lateral, and protrusive excursions.

### 2.3. Follow-Up and Data Collection

The silicon replicas were sectioned, and the thickness of the light-body silicone will be measured using a stereomicroscope. This thickness represented the marginal and internal misfit of the laminate veneers. Treatment-related data, including baseline information, oral hygiene, proximal contact breakage, enamel preservation, and endodontic treatment status, were also collected.

The patients were re-evaluated two weeks after restoration placement (T1) for final excessive resin removal and occlusal adjustment. Subsequent evaluations occurred at six-month (T2) and one-year (T3) follow-ups. In these appointments, a calibrated examiner assessed the periodontal health and veneer performance. The periodontal health was evaluated, including plaque accumulation, gingival appearance, spontaneous bleeding, and periodontal pocket depth (PPD) measurement [19]. Specifically, the plaque index (0–3 score) assessed plaque by running a probe along the abutment surface at the gingival sulcus, with scores ranging from 0 (no plaque) to 3 (signifying heavy, visible plaque), and the gingival index evaluated inflammation, with scores from 0 (healthy gingiva) to 3 (severe inflammation characterized by redness, swelling, ulcers, and spontaneous bleeding). The veneer performance was scored according to the modified United States Public Health Service (USPHS) criteria, including shade, contour, marginal adaptation, fracture, detachment, and tooth hypersensitivity [19]. These criteria were graded from alfa (A) if there was no problem, bravo (B) if a small complication resulted, charlie (C) if a major complication presented, to delta (D) if veneer removal was required (Table 1). A second examiner also measured fit, USPHS criteria, and periodontal indices on 20% of the restorations to assess inter-examiner reliability. Additionally, the patients rated their overall satisfaction using a 100 mm visual analog scale (VAS), where 0 represented extreme dissatisfaction and 100 represented extreme satisfaction. Furthermore, patients were provided with detailed oral hygiene instructions at baseline and at each follow-up, including the use of non-abrasive toothpaste, daily flossing, and avoidance of habits such as hard-object biting.

### 2.4. Outcome Measurements

The primary outcome was the one-year survival and success of the laminate veneers, assessed by the modified USPHS criteria. Success was defined as the absence of veneer detachment (even if it can be recemented), ceramic fracture (even repairable), biological complications, and the need for restoration replacement due to morphology or shade mismatch or marginal misfit. For vital teeth, success also included no tooth sensitivity and maintained pulp vitality, confirmed with pulp tests. For endodontically treated teeth, success was defined as the absence of spontaneous pain, sensitivity to percussion, dental mobility, abscess, and fistula.

The secondary outcomes comprised proper restoration fit with marginal and internal gaps lower than 120 µm, healthy gingiva, no plaque accumulation, a periodontal pocket depth within the normal range of 3 mm, and high patient satisfaction [18].

### 2.5. Statistical Analysis

All statistical analyses were performed with statistical software (SPSS ver. 22, IBM, Armonk, NY, USA). The demographic features of the two groups were compared using the Pearson chi-square test. The intraclass correlation coefficient (ICC) was employed to analyze inter-examiner reliability of fit measurements, USPHS grades, and periodontal indexes. Kaplan-Meier survival analysis and log-rank test were used to assess and compare the survival and success rates of the two groups. Student’s independent *t*-test was utilized to compare internal and marginal gaps between the two groups. The Mann-Whitney U test was used to compare the USPHS grades, periodontal indexes, and satisfaction VAS scores between the two groups at each time point. The differences between USPHS grades, periodontal indexes, and satisfaction VAS scores at different follow-up times within each group will be evaluated with the Friedman test, followed by post hoc Wilcoxon signed-rank tests with Bonferroni correction to identify specific differences.

## 3. Results

### 3.1. Patient Demographics

In total, 52 patients were enrolled, with 26 patients assigned to the zirconia (ZV) group and 26 to the lithium disilicate (LDV) group. One patient in the LDV group was lost to follow-up at the six-month mark, and thus, data from that patient were excluded from the corresponding analysis. The baseline characteristics of the two groups were similar in terms of age, sex, tooth type, and oral hygiene habits (Table 2). No significant differences were observed between the two groups regarding preparation characteristics, such as incisal thickness, labial thickness, and enamel preservation.

### 3.2. Primary Outcomes

**Figure 4:**
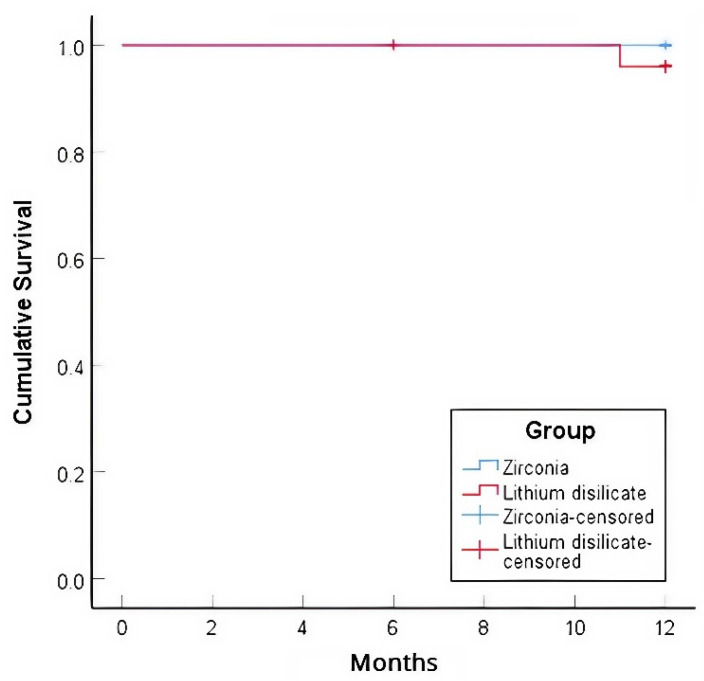
Kaplan–Meier survival curves of lithium disilicate and zirconia veneers after 12 months.

In terms of the primary outcome, the one-year Kaplan-Meier survival rate for zirconia veneers was 100%, while for lithium disilicate veneers, it was 96.0% (Figure 4). One veneer in the zirconia group debonded at one month but was successfully rebonded (still accounted as survival); this involved a central incisor, a vital tooth, with no proximal breakage, and occurred in a male patient with an edge-to-edge bite. After rebonding, careful occlusal adjustment was performed to reduce excessive incisal loading, and the veneer remained functional and intact for the remainder of the study period without further complications. In the lithium disilicate group, one veneer debonded at 11 months, accompanying a fracture, and was replaced with a new veneer; this involved a mandibular second premolar, which had been endodontically treated, with no proximal breakage. The log-rank test showed no statistically significant difference in survival rates between the groups (χ^2^ = 1.04, *p* = 0.308). The success rate for zirconia veneers, accounting for the debonding that could be rebonded, was 96.2%, whereas the success rate for lithium disilicate veneers was 96.0%.

### 3.3. Secondary Outcomes

High ICCs ranging from 0.911 to 0.934 for fit, 0.807 to 1 for USPHS, and 0.814 to 0.966 for periodontal indexes confirmed strong inter-examiner reliability. The marginal and internal gap measurements showed no significant difference between the zirconia and lithium disilicate veneer groups. The zirconia group had an average marginal gap of 72.35 ± 11.60 µm, and the lithium disilicate group had 74.09 ± 12.57 µm (*p* = 0.605). For the internal gap, zirconia averaged 102.15 µm ± 26.07 µm, and lithium disilicate 98.06 µm ± 20.41 µm, also showing no significant difference (*p* = 0.532).

The Mann-Whitney U test demonstrated no statistically significant intergroup differences in any of the modified USPHS criteria assessed, namely contour, shade, marginal adaptation, fracture, retention, and tooth hypersensitivity (*p* > 0.05). Both groups exhibited excellent performance, characterized by high frequencies of alfa and bravo grades (Table 3). However, the zirconia group showed slightly higher frequencies of alfa scores for contour at all time points, while the lithium disilicate group presented slightly more alfa scores for shade. Friedman’s test revealed no significant changes in performance over time for any of the evaluated criteria (*p* > 0.05). Nonetheless, a small gradual increase in bravo ratings was noted for some criteria, particularly marginal adaptation.

Regarding periodontal health, Mann-Whitney tests revealed no significant differences in plaque and gingival index scores between the study groups at any time point (*p* > 0.05) (Table 4). Furthermore, the Friedman test revealed no significant differences in plaque and gingival index scores among the follow-up time points when comparing within each ceramic type. Similarly, the PPD measurements also showed no significant differences between the study groups at any time point (*p* > 0.05) and no significant changes within each ceramic type over the follow-up period (*p* > 0.05) (Table 5). Patient satisfaction, assessed using VAS, showed a significant increase over time within the LDV group (*p* = 0.006), while the ZV group maintained consistently high satisfaction scores without significant changes (*p* = 0.160). However, there were no significant differences in patient satisfaction scores between the LDV and ZV groups at any time point (*p* > 0.05).

## 4. Discussion

The findings of this study do not support the rejection of the null hypothesis, as there was no statistically significant difference in the one-year clinical performance between acid-etched zirconia laminate veneers and lithium disilicate veneers. Both materials demonstrated comparable survival and success rates, with zirconia veneers exhibiting a 96.2% success rate and lithium disilicate veneers showing a 96.0% success rate. The absence of significant differences in veneer retention, fracture resistance, marginal adaptation, periodontal indexes, and patient satisfaction suggests that zirconia laminate veneers perform on par with lithium disilicate veneers. A comparison with the three-year RCT of Fawakhiri et al. evaluating high-translucency cubic zirconia and lithium disilicate veneers reveals consistent findings regarding the clinical performance of both materials [13]. The three-year study reported no significant differences between the two groups across esthetic, functional, and biological parameters, with lithium disilicate showing slightly better translucency and esthetics. Additionally, the RCT observed a minor increase in hypersensitivity in the lithium disilicate group, whereas in the current study, hypersensitivity levels were comparable between the two groups, with no significant difference detected. The congruence between our one-year findings and the three-year results further supports zirconia veneers as a durable and esthetically acceptable option alongside lithium disilicate veneers.

The survival and success rates observed in the current study align with those reported in previous literature evaluating different ceramic veneer materials. The meta-analysis by Klein et al. reported a pooled survival rate of 96.81% for lithium disilicate veneers over 10.4 years, which closely matches the 96.0% survival rate found in this study at the one-year follow-up [20]. Comparatively, feldspathic and leucite-reinforced glass-ceramic veneers demonstrated slightly lower survival rates of 96.13% and 93.70%, respectively, with higher complication rates than lithium disilicate. The long-term study by Beier et al. further reinforced the durability of silicate ceramic veneers, reporting a survival rate of 94.4% at five years, 93.5% at ten years, and 82.93% at twenty years, with ceramic fractures being the most common mode of failure [21]. Similarly, a retrospective study by Layton et al. estimated a ten-year survival rate of 96.0% for feldspathic porcelain veneers, while Guess et al. reported a five-year survival rate of 97.5% for overlap veneers, demonstrating the reliability of ceramic materials over time [22,23]. These findings indicate that zirconia laminate veneers with acid-etched surface treatment, as evaluated in this study, achieve a comparable short-term survival rate to lithium disilicate and other traditional ceramic veneers.

In this study, both zirconia and lithium disilicate veneers experienced debonding, but their failure modes differed significantly. A zirconia veneer detached early within the first month, whereas a lithium disilicate veneer debonded later at 11 months but fractured upon failure. The early debonding of the zirconia veneer may be attributed to possible contamination during the porcelain layering or bonding process, which could have compromised the adhesive interface. Additionally, the patient’s occlusion might have played a role, as edge-to-edge bite relationships exert excessive stress on ceramic veneers during function [24]. Fortunately, in this case, the zirconia veneer remained intact without porcelain chipping, likely due to the high elastic modulus of the zirconia coping, which helps prevent tensile stress generation within the porcelain and allows for rebonding [25]. A key challenge in rebonding the zirconia veneer was the loss of its initial dull intaglio surface texture due to contamination, as re-etching with the acid mixture was not an option due to the potential risk of damaging the layered porcelain. Instead, a sandblasting protocol was employed to recreate micro-retentive features for bonding. The successful service of this veneer for the remaining 11 months indicates that surface reconditioning can restore adequate bonding strength, provided the veneer remains structurally intact. In contrast, the debonded lithium disilicate veneer not only occurred later but also resulted in fracture, necessitating full replacement. This highlights a key difference between the two materials, while lithium disilicate provides excellent bondability, its inferior fracture resistance makes it more susceptible to catastrophic failure upon debonding. This aligns with findings from previous studies, where lithium disilicate veneers demonstrated a higher risk of failure due to cohesive fractures when subjected to high-stress occlusal conditions [21].

Surface treatment of zirconia remains a topic of ongoing debate, with various methods showing different outcomes in terms of bond strength and material integrity. Sandblasting with aluminum oxide is widely used to increase surface roughness and enhance micromechanical retention, but it may introduce surface defects and increase monoclinic phase content, potentially reducing flexural strength [26]. More recently, chemical etching using a mixture of hydrofluoric and nitric acids has emerged as a viable alternative, producing microroughness without abrasive blasting and enabling chemical bonding via phosphate groups in MDP-based adhesives. However, while laboratory data are promising, long-term clinical evidence is still limited, and further validation in dynamic oral environments is warranted. In this study, the zirconia surface was etched with a hydrofluoric acid-nitric acid mixture and bonded using a light-cured polymeric resin cement containing MDP. This adhesive strategy was selected based on its proven chemical affinity for metal oxides and its superior bond strength compared to sandblasting [12]. Additionally, a light-cured system was preferred over dual-cure cements to minimize color instability, as it avoids the use of tertiary amines, common initiators in dual-cure systems that can lead to discoloration over time. This consideration is particularly critical in esthetically demanding anterior regions.

In the in vitro study by Anh et al., the shear bond strength of acid-etched zirconia (19.85 MPa) to light-cure veneer adhesive was found to be higher than that of lithium disilicate (16, 26 MPa) [12]. However, the clinical oral environment may present different challenges due to temperature fluctuations and dynamic loading. The thermal expansion coefficient of lithium disilicate (11.4 × 10^−6^/°C) is slightly closer to that of dental composite polymer (22.5 × 10^−6^/°C) and tooth enamel (16.96 × 10^−6^/°C), resulting in less stress at the adhesive interface during temperature changes [27,28,29]. This closer compatibility may contribute to more stable long-term bonding and reduced risk of adhesive failure over time. In contrast, zirconia’s lower thermal expansion (10.8 × 10^−6^/°C) compatibility with composite resin could lead to increased interfacial stress under cyclic thermal conditions, potentially affecting long-term bond durability [30]. In terms of mechanical behavior, zirconia demonstrates markedly superior flexural strength (800–1200 MPa) and fracture toughness (6–8 MPa·m½) compared to lithium disilicate (360–440 MPa and 2.5–3.0 MPa·m½, respectively) [31]. These material characteristics enhance zirconia’s resistance to crack propagation and fracture under load, particularly in thin veneer designs or high-stress zones. In contrast, lithium disilicate has lower mechanical strength, predisposing it to cohesive failure under functional stress.

The insignificant reduced shade match of zirconia veneers compared to lithium disilicate veneers is in line with Kim et al., who noted that while increased yttria content enhances zirconia’s optical properties, it still falls short of the translucency achieved by lithium disilicate [32]. The difference may stem from zirconia’s polycrystalline structure, which scatters light more than the glass-based composition of lithium disilicate. Nonetheless, zirconia’s superior masking ability remains advantageous for cases requiring coverage of underlying discoloration. The sensitivity reduction observed over time in both groups was consistent with Nejatidanesh et al., who highlighted the role of bonded restorations in minimizing postoperative sensitivity [1]. The absence of significant inflammation and increased probing depths suggests that proper marginal adaptation minimizes plaque accumulation and supports periodontal stability. Additionally, patient satisfaction tended to increase over time, aligning with the findings of Moura et al., who reported that long-term adaptation and esthetic integration contribute to improved patient-reported outcomes [19].

The marginal and internal fit of veneers significantly impacts their longevity, influencing microleakage, secondary caries, and retention. The comparable and clinically acceptable fit of milled zirconia and lithium disilicate veneers in this study aligns with findings from Nguyen et al. and Anh et al. [33,34]. Their studies confirmed no significant differences between these two materials in trueness, precision, marginal, and internal adaptation when fabricated via CAD/CAM milling. The accuracy of both materials reinforces the reliability of CAD/CAM manufacturing, making them suitable for long-term clinical use.

Despite its clinically relevant findings, this study has several limitations. First, the one-year follow-up may not fully capture long-term failure modes, such as marginal degradation, secondary caries, or bond deterioration over time. The sample size, while relatively small, was calculated based on prior clinical data and ethical considerations related to randomized controlled trials in prosthodontics, where interventions are irreversible. Moreover, our trial strictly followed the International Committee of Medical Journal Editors guidelines, including registration on ClinicalTrials.gov, ensuring transparency and prospective reporting. While some previous studies have longer follow-up periods, they are mostly retrospective; notably, the only existing 3-year RCT on this topic was not registered on clinical trial platforms [1,13,22,23]. Extended longitudinal research with larger samples and registered protocols is needed to confirm and expand upon our findings. Second, while shade stability was evaluated at three time points, extended follow-ups are needed to determine color changes due to aging, staining, or material degradation. Third, although all clinical procedures were performed by a single calibrated operator and restorations were fabricated in the same laboratory using standardized CAD/CAM technology, the potential influence of operator skill, laboratory precision, and subtle variations in bonding protocol execution should be acknowledged. Standardization and reporting of these procedural variables are essential for enhancing research reproducibility and clinical relevance. Fourth, patient-specific factors such as occlusal forces, parafunctional habits, and oral hygiene variations could influence long-term outcomes, which were not fully accounted for in this study. Additionally, the study did not explore the impact of different surface treatments on the bonding longevity of zirconia veneers, which remains a critical consideration for optimizing their clinical performance. Finally, although multilayer and gradient zirconia materials are gaining attention, their clinical performance in minimal-thickness veneers remains under-investigated. Future research should assess the effects of bonding protocols, occlusal forces, and patient-specific variables on the survival and success of zirconia and lithium disilicate veneers, while also exploring alternative bonding systems and advanced material generations to enhance their applicability in esthetically demanding cases.

## 5. Conclusions

This randomized, double-blind controlled clinical trial supports the short-term clinical viability of acid-etched zirconia laminate veneers as a comparable alternative to lithium disilicate veneers. Both materials demonstrated high survival rates and comparable esthetic and functional outcomes, with no statistically significant differences in marginal adaptation, periodontal response, or hypersensitivity. The use of MDP-containing polymeric adhesive systems contributed to stable bonding despite intrinsic differences in material composition. Notably, this study focused on single-unit restorations, where esthetic integration is more technically demanding, and represents the first RCT evaluating zirconia veneers etched with a hydrofluoric-nitric acid mixture. While these findings are promising, the limited follow-up duration and sample size warrant cautious interpretation. Future research involving larger cohorts, longer observation periods, and emerging material systems such as multilayered zirconia is necessary to validate and expand upon these results.

## Figures and Tables

**Table 1:** Laminate veneer evaluation according to the modified United States Public Health Service (USPHS) criteria.

Category	Alfa	Bravo	Charlie	Delta
Contour	No mismatch with contralateral tooth	Little morphology difference with contralateral tooth	Moderate morphology difference with contralateral tooth	Veneer requiring replacement
Shade	No mismatch with adjacent teeth	Litter color or translucency difference with adjacent tooth	Moderate color or translucency difference with adjacent tooth	Veneer requiring replacement
Marginal adaptation	No probe catch	Mild probe catch without marginal gap	Marginal gap existing	Marginal chipping or veneer missing
Fracture	No fracture	Ceramic chipping without impairing aesthetics or function	Ceramic chipping with exposed tooth structure but repairable	Major fracture require replacement
Detachment	No debonding	Debonding, may be re-bonded	Repeated debonding after rebonding	Debonding and fracture, cannot be re-bonded
Tooth hypersensitivity	No hypersensitivity under airflow	Hypersensitivity disappears after stopping airflow	Hypersensitivity persists after stopping airflow	Spontaneous pain

**Table 2:** Baseline demographic and clinical characteristics of the control and experimental groups.

Variable		Group		*p*
		Control	Experimental	
Age (years)		29.0 ± 6.9	32.3 ± 12.4	0.253
Sex	Male	6 (23.1%)	3 (11.5%)	0.271
	Female	20 (76.9%)	23 (88.5%)	
Tooth type	Anterior	11 (42.3%)	13 (50%)	0.578
	Posterior	15 (57.7%)	13 (50%)	
Incisal thickness (mm)		1.35 ± 0.30	1.50 ± 0.30	0.316
Labial thickness (mm)		0.64 ± 0.06	0.65 ± 0.09	0.056
Proximal breakage		3 (11.5%)	4 (15.4%)	0.685
Endodontic treatment		16 (61.5%)	19 (73.1%)	0.375
Enamel preservation	Entire enamel	7 (26.9%)	5 (19.2%)	0.510
	≥70% enamel	19 (73.1%)	21 (80.8%)	
Oral hygiene	Brush and floss twice a day	1 (3.8%)	3 (11.5%)	0.570
	Brush twice a day	17 (65.4%)	15 (57.7%)	
	Brush once a day	8 (30.8%)	8 (30.8%)	

**Table 3:** Veneer performance according to modified United States Public Health Service (USPHS) criteria at 2 weeks (T1), 6 months (T2), and 12 (T3) months.

Indexes	Lithium Disilicate n (%)				Zirconia n (%)				P^1^
	A	B	C	D	A	B	C	D	
Contour									
T1	17 (65.4)	9 (34.6)			21 (80.8)	5 (19.2)			0.216
T2	16 (64.0)	9 (36.0)			21 (80.8)	5 (19.2)			0.184
T3	14 (58.3)	10 (41.7)			19 (73.1)	7 (26.9)			0.276
P^2^	0.368				0.135				
Shade									
T1	14 (53.8)	12 (46.2)			8 (30.8)	18 (69.2)			0.095
T2	12 (48.0)	13 (52.0)			8 (30.8)	18 (69.2)			0.212
T3	11 (45.8)	13 (54.2)			7 (26.9)	19 (73.1)			0.168
P^2^	0.223				0.368				
Marginal adaptation									
T1	21 (80.8)	5 (19.2)			22 (84.6)	4 (15.4)			0.717
T2	19 (76.0)	6 (24.0)			21 (80.8)	5 (19.2)			0.682
T3	18 (75.0)	6 (25.0)			19 (73.1)	7 (26.9)			0.878
P^2^	0.223				0.097				
Fracture									
T1	25 (96.2)	1 (3.8)			26 (100)				0.317
T2	24 (96.0)	1 (4.0)			26 (100)				0.308
T3	23 (92.0)	1 (4.0)		1 (4.0)	26 (100)				0.145
P^2^	0.368				–				
Detachment									
T1	26 (100)				26 (100)				–
T2	25 (100)				25 (96.2)	1 (3.8)			0.327
T3	24 (96.0)			1 (4.0)	25 (96.2)	1 (3.8)			0.955
P^2^	0.368				0.368				
Hypersensitivity									
T1	24 (92.3)	2 (7.7)			24 (92.3)	2 (7.7)			–
T2	25 (100)				26 (100)				–
T3	24 (100)				26 (100)				–
P^2^	0.368				0.135				

P^1^ Significance level for USPHS score comparison regarding performance between groups according to Mann-Whitney tests; P^2^ Significance level for comparison within the same group among time points according to Friedman tests.

**Table 4:** Plaque (PI) and gingival index (GI) scores of veneer groups at 2 weeks (T1), 6 months (T2), and 12 (T3) months.

Indexes	Lithium Disilicate n (%)				Zirconia n (%)				P^1^
	0	1	2	3	0	1	2	3	
PI									
T1	14 (53.8)	12 (46.2)			18 (69.2)	8 (30.8)			0.259
T2	12 (48.0)	10 (40.0)	3 (12.0)		17 (65.4)	7 (26.9)	2 (7.7)		0.223
T3	11 (15.8)	9 (37.5)	4 (16.7)		16 (61.5)	8 (30.8)	2 (7.7)		0.225
P^2^	0.092				0.115				
GI									
T1	15 (57.7)	11 (42.3)			17 (65.4)	9 (34.6)			0.572
T2	13 (52.0)	11 (44.0)	1 (4.0)		15 (57.7)	10 (38.5)	1 (3.8)		0.698
T3	12 (50.0)	10 (41.7)	2 (8.3)		16 (61.5)	8 (30.8)	2 (7.7)		0.615
P^2^	0.405				0.197				

P^1^ Significance level for periodontal index comparison regarding performance between groups according to Mann-Whitney tests; P^2^ Significance level for comparison within the same group among time points according to Friedman tests.

**Table 5:** Probing pocket depth (PPD) and visual analog scale (VAS) scores for lithium disilicate and zirconia restorations at 2 weeks (T1), 6 months (T2), and 12 (T3) months.

	Lithium Disilicate				Zirconia				P^1^
	M	Min–Max	Q25	Q75	M	Min–Max	Q25	Q75	
PPD (mm)									
T1	2.00 ^a^	1.00–2.83	1.63	2.54	2.00 ^a^	1.00–2.83	1.50	2.67	0.811
T2	2.17 ^a^	1.00–3.33	1.33	2.67	2.17 ^a^	1.17–3.33	1.63	2.71	0.532
T3	2.00 ^a^	1.00–3.50	1.17	2.46	2.08 ^a^	1.00–3.33	1.50	2.33	0.807
P^2^	0.726				0.104				
VAS									
T1	82.5 ^a^	70.0–100.0	75.0	86.3	77.5 ^a^	70.0–100.0	75.0	80.0	0.079
T2	85.0 ^b^	70.0–100.0	80.0	95.0	80.0 ^a^	65.0–100.0	75.0	90.0	0.111
T3	87.5 ^b^	65.0–100.0	80.0	100.0	82.5 ^a^	65.0–100.0	75.0	85.0	0.060
P^2^	0.006				0.160				

P^1^ Significance level for PPD and VAS comparison regarding performance between groups according to Mann-Whitney tests; P^2^ Significance level for comparison within the same group among time points according to Friedman tests. Different superscript lowercase letters indicate significant differences between time points according to Wilcoxon signed-rank tests.

## Data Availability

The data presented in this study are available on request from the corresponding author due to privacy restrictions.

## Abbreviations

The following abbreviations are used in this manuscript:

3D	Three-dimensional
CAD/CAM	Computer-aided design/manufacturing
CONSORT	Consolidated standards of reporting trials
LDV	Lithium disilicate veneer
MDP	10-methacryloyloxydecyl dihydrogen phosphate
PPD	Probing pocket depth
RCT	Randomized clinical trial
USPHS	United States Public Health Service
VAS	Visual analog scale
ZV	Zirconia veneer
